# Beyond the Typical Papule: A Case Report and Review of a Peptoniphilus asaccharolyticus Abscess in Molluscum Contagiosum

**DOI:** 10.7759/cureus.86941

**Published:** 2025-06-28

**Authors:** Shivani Wadhwa, Sanjeev Gupta, Aditi Dabhra

**Affiliations:** 1 Department of Dermatology, Fakeeh University Hospital, Dubai, ARE; 2 Dermatology, Maharishi Markandeshwar (MM) Institute of Medical Sciences and Research (Deemed to be University), Ambala, IND

**Keywords:** anaerobic, cutaneous abscess, molluscum contagiosum, peptoniphilus asaccharolyticus, secondary bacterial infection

## Abstract

Molluscum contagiosum (MC) is a commonly encountered viral skin infection caused by a poxvirus, primarily affecting children, sexually active adults, and immunocompromised individuals. It typically presents as small, dome-shaped, flesh-colored papules with central umbilication. In most cases, the condition is benign, self-limiting, and resolves spontaneously over several months without the need for aggressive treatment. However, complications such as secondary bacterial infection or abscess formation are uncommon, particularly in immunocompetent adults. When such complications do arise, they can pose significant diagnostic and therapeutic challenges, especially when the presentation mimics more common cutaneous infections such as furuncles or other bacterial abscesses. This case report discusses an unusual presentation of molluscum contagiosum in an immunocompetent adult, where the lesion evolved into a deep-seated cutaneous abscess. The atypical clinical features initially obscured the diagnosis, raising concerns for more common pyogenic infections. This underscores the necessity for clinicians to maintain a broad differential diagnosis when encountering atypical skin lesions and highlights the importance of performing appropriate microbial cultures, histopathological examinations, and targeted antimicrobial therapy.

## Introduction

Molluscum contagiosum (MC) is a viral skin infection caused by a double-stranded DNA virus that is a member of the Poxviridae family [1]. It typically presents as small, dome-shaped, umbilicated papules and is usually self-limiting in immunocompetent individuals. After inoculation, the MC virus regulates host cell transcription and replicates within the cytoplasm. As the virus-laden cells mature, they become larger and move toward the surface. Viral particles eventually displace the host cell organelles and nucleus, and infected cells develop the characteristic Henderson-Paterson bodies [2]. However, secondary bacterial infection and cutaneous abscess formation are uncommon but clinically significant complications.

In the present case, we report a deep-seated cutaneous abscess secondary to an MC lesion in an immunocompetent adult, wherein *Peptoniphilus (P.) asaccharolyticus*, a gram-positive, obligate anaerobic coccus, was isolated as the sole pathogen. This finding is of particular interest because Peptoniphilus species, although commensals of human skin, gut, and genitourinary tracts, are rarely reported as primary pathogens in skin and soft tissue infections.

Only isolated case reports exist describing *P. asaccharolyticus* as a causative agent in infections such as breast abscesses, necrotizing fasciitis, and bacteremia, typically in immunocompromised hosts or those with prior breaches in skin integrity. The occurrence of a monomicrobial Peptoniphilus abscess in an otherwise healthy individual, in the context of MC, is thus exceptional and highlights the evolving spectrum of anaerobic skin pathogens.

While MC generally resolves spontaneously over several months, treatment may be warranted in cases of cosmetic concern, extensive involvement, immunosuppression, or secondary complications. Management options include physical modalities such as curettage and cryotherapy, and topical agents like potassium hydroxide. In cases complicated by secondary bacterial infection, targeted antimicrobial therapy based on culture sensitivity becomes essential.

## Case presentation

**Figure 1:**
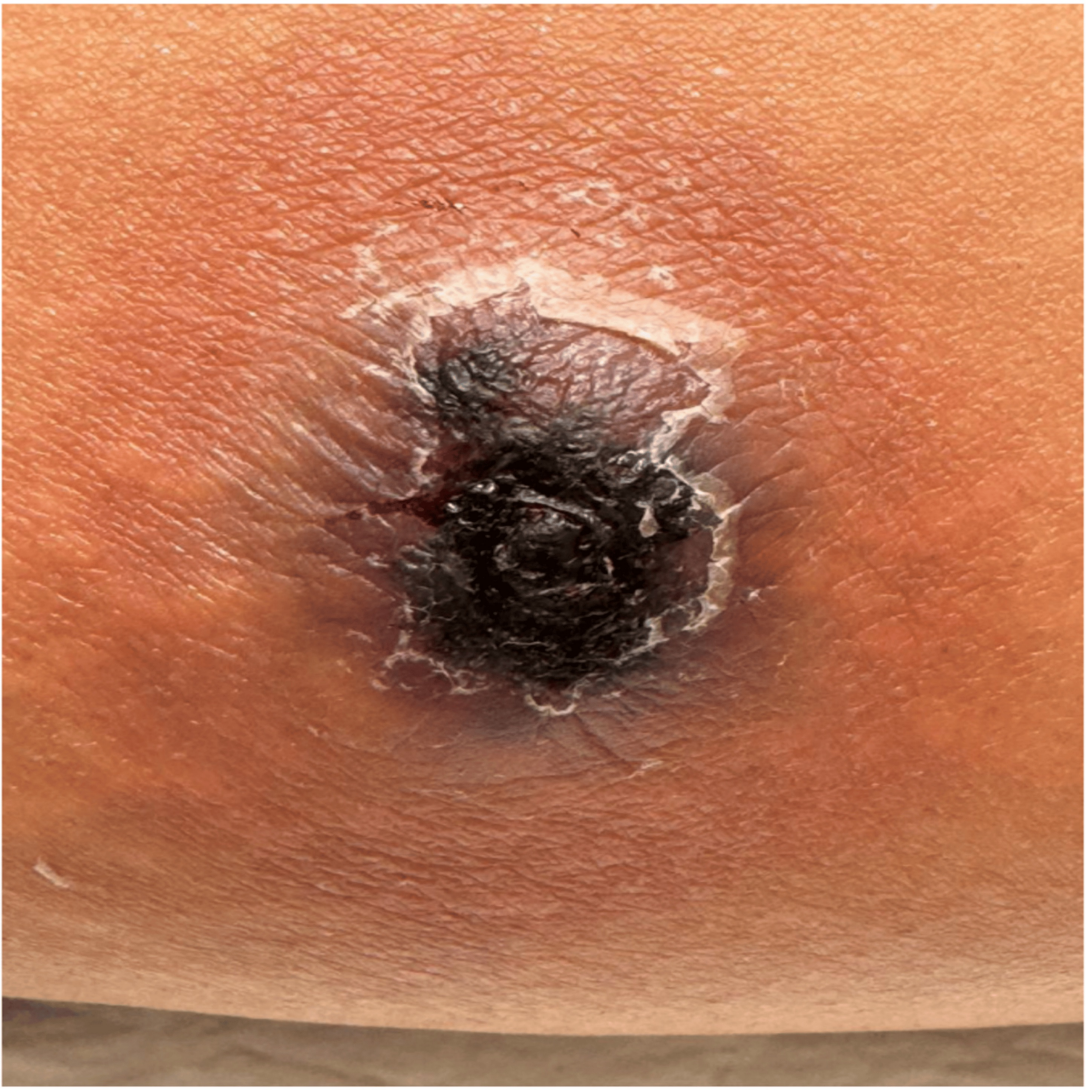
An erythematous, tender, fluctuant swelling measuring approximately 4-5 cm, with necrotic slough at the center

An immunocompetent 34-year-old female presented to our dermatology outpatient department with complaints of redness and swelling over the inner part of the left thigh for the past four days. She reported a history of a small, painless lesion at the same site for approximately a year, which had become painful and swollen in the last four days. There were no associated systemic symptoms or history of trauma. On examination, an ill-defined, tender, erythematous nodular lesion measuring approximately 1.5 cm was noted on the medial aspect of the left thigh. The lesion was firm on palpation with no fluctuation. Based on the clinical presentation, the differential diagnoses included an infected sebaceous cyst and a furuncle. The patient was initially treated with topical and systemic antibiotics (mupirocin ointment and amoxicillin-clavulanate tablets for their broad-spectrum role against common skin pathogens) along with nonsteroidal anti-inflammatory drugs (NSAIDs) for five days. Upon follow-up, the patient reported persistent pain and an increase in the lesion’s size. On re-examination, the lesion had progressed to an erythematous, tender, fluctuant swelling measuring approximately 4 cm x 5 cm, with necrotic slough at the center (Figure 1).

The patient remained afebrile throughout the course of the infection. A diagnosis of a cutaneous abscess was made, and incision and drainage were performed under sterile conditions. Pus was collected and sent for culture and sensitivity testing, which identified *P.* *asaccharolyticus* as the only organism grown that was sensitive to metronidazole.

**Figure 2:**
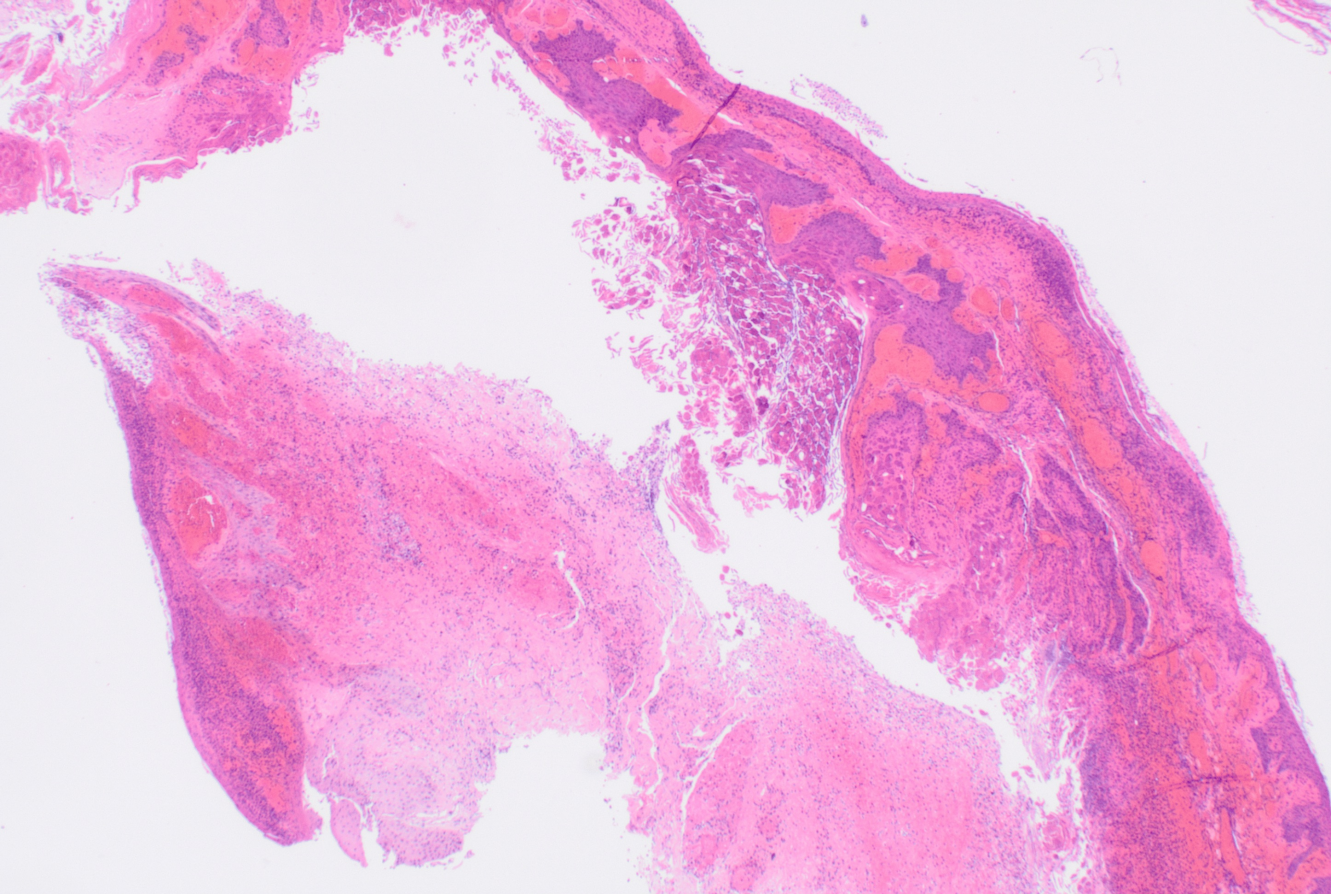
Histopathological examination that showed fragments of skin in which the epidermis incorporated molluscum contagiosum Hematoxylin and eosin stain, 10x10x

**Figure 3:**
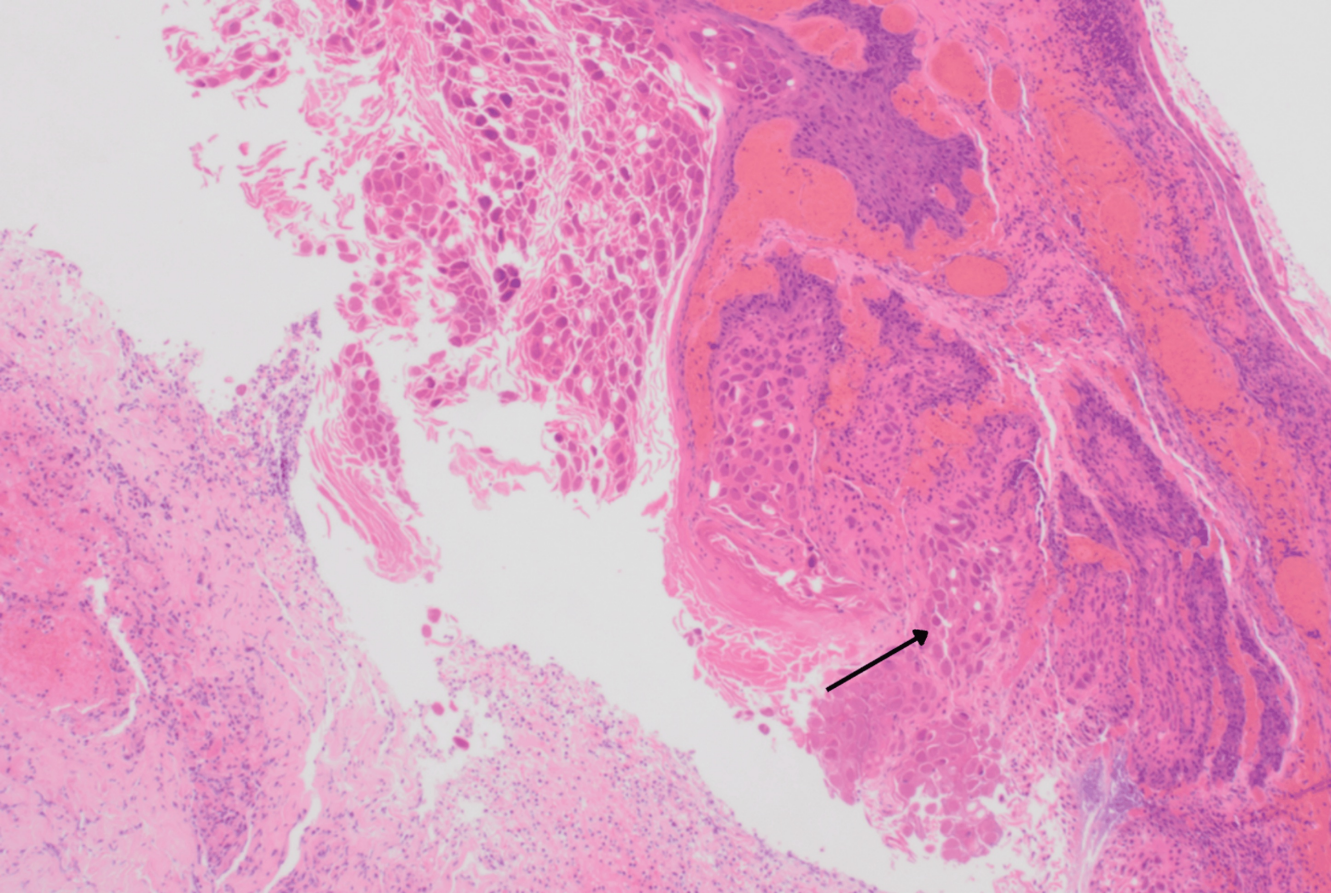
Histopathological examination showing edematous and inflamed dermis with characteristic molluscum bodies (black arrow) Hematoxylin and eosin, 10x100x

The excised tissue was sent for a histopathological examination that showed fragments of skin in which the epidermis incorporated molluscum contagiosum (Figure 2) and edematous and inflamed dermis with microabscess formation (Figure 3).

Viral markers, including human immunodeficiency virus (HIV), hepatitis B surface antigen (HBsAg), and hepatitis C virus (HCV), were negative. Other investigations, like complete blood count and random blood sugar, were within the normal range. The patient was prescribed metronidazole tablets for seven days and showed complete resolution of symptoms within two weeks. Two months later, the patient presented with a typical molluscum contagiosum lesion in the pubic region, which was extracted and healed without complications. This case highlights the importance of recognizing secondary bacterial infections in molluscum contagiosum, considering anaerobic pathogens in non-resolving skin infections, and tailoring antibiotic therapy based on culture results.

## Discussion

MC and cutaneous abscess formation: diagnostic challenges

MC is a common cutaneous viral infection caused by a double-stranded DNA virus belonging to the Poxviridae family. It primarily infects the epidermis, resulting in dome-shaped, umbilicated papules with an average diameter of 3-5 mm [1]. The infection spreads via direct skin-to-skin contact or through contaminated objects. Diagnosis is primarily clinical, based on the characteristic papules, but can be confirmed via biopsy and histopathology when necessary [2]. In immunocompetent individuals, MC lesions typically resolve spontaneously over time [1,2]; however, in immunocompromised patients, the lesions tend to be more severe, widespread, and persistent [1].

While MC is usually a benign and self-limiting condition, secondary bacterial infections can complicate its course, particularly when lesions are manipulated or exposed to trauma. Abscess formation, though rare, can occur due to the discharge of molluscum bodies into the dermis, leading to local inflammation, neutrophilic infiltration, and subsequent abscess development [3,4]; this inflammatory response can further be complicated or associated with secondary bacterial infection.

Most reported cases of MC-associated abscesses involve children with or without immunodeficiency or HIV infection [5-7,8-10]. These reports in immunocompetent hosts have most commonly been seen involving periorbital areas in children [8-10]; such reports are rarely reported in immunocompetent adults.

Shaham et al. reported a case series of 10 children with MC presenting as periorbital abscesses [8]. Brandrup and Asschenfeldt reported a case of a nine-year-old boy who had recurrent fluctuating abscesses of the forearm caused by repeated squeezing of the comedo-like lesions caused by molluscum contagiosum [11]. The presence of other typical lesions of MC in these cases was helpful to make the diagnosis, but making such a diagnosis can be very challenging in the cases of solitary lesions presenting as a cutaneous abscess, as in our case.

Diagnostic workup for secondary infections in MC

Secondary bacterial infections of MC lesions are uncommon [12]. Diagnosing secondary infections in MC requires a thorough clinical evaluation, particularly when lesions exhibit atypical signs such as inflammation, tenderness, or rapid enlargement.

While *Staphylococcus aureus* and *Streptococcus pyogenes* are the most common pathogens associated with secondary bacterial infections of MC [13], this case presents an unusual causative organism, *P. asaccharolyticus, *which are Gram-positive anaerobic cocci (GPAC) and a member of the Peptostreptococcaceae family in the phylum Firmicutes [2]. It produces butyric acid as the major metabolic end product and uses peptone and amino acids, rather than carbohydrates, as its major energy sources, hence the name 'Peptoniphilus' (peptone + philic). There are no cases of *Peptoniphilus* infection of molluscum reported in the literature.

GPAC usually colonize the human skin, mouth, upper respiratory tract, gut, and genitourinary tract as commensals, constituting part of the human normal microbiota, but can act as opportunistic pathogens, particularly in deep-seated soft tissue infections, abscesses, and post-surgical wounds [14]. Other diseases caused by this group of bacteria include skin and soft tissue infections, bone and joint infections (including infections of prosthetic joints), osteomyelitis, arthritis, bloodstream infections, urinary tract infections, pulmonary pneumatocele, peritonsillar abscess, renal abscess, bacterial vaginosis, meningitis, pericarditis, scrotal abscess, and spinal abscess [15].* P. asaccharolyticus *is typically associated with polymicrobial infections, but its potential as a virulent pathogen in monomicrobial invasive infections has been underestimated. A total of seven cases of mono-infection have been reported in the literature [15].

The initial misdiagnosis of the lesion as an infected sebaceous cyst or furuncle highlights the diagnostic challenge posed by secondary infections of MC. The histopathological examination of the excised tissue from the left inner thigh provided crucial insights into the nature of the lesion. The microscopic analysis confirmed the presence of molluscum contagiosum, characterized by epidermal involvement with molluscum bodies. Additionally, the dermis exhibited edema, inflammation, and microabscess formation, reinforcing the diagnosis of a secondary bacterial infection. Importantly, there was no evidence of dysplasia or malignancy, ruling out other concerning differentials. Culture and sensitivity testing of the pus helped in identifying the pathogen and in guiding appropriate antimicrobial therapy.

Management strategies for MC with secondary infection

Standard treatment for MC includes observation, curettage, cryotherapy, and topical agents such as imiquimod and potassium hydroxide [16]. However, surgical treatment is generally recommended for abscesses associated with molluscum [8]. Shaham et al., in a case series of 10 immunocompetent children with periorbital molluscum-associated abscesses, reported that these abscesses had a suboptimal response to antibiotics, and almost all cases needed surgery and intraoperative cryotherapy for recovery; culture sensitivity was done only in 6 cases and was reported to be positive in only 2 of them [8]. Our patient did not show any significant improvement with initial antibiotics, but improved after the surgical intervention; clinical outcome further improved after the initiation of targeted antibiotic therapy according to the culture sensitivity. All these reported cases of molluscum contagiosum are summarized in Table 1.

**Table 1:** Reported cases of molluscum contagiosum associated with cutaneous abscess formation C&S: culture and sensitivity

Study / Author	Year	Age/Sex	Immune Status	Site Involved	Causative Organism	Treatment	Outcome
Brandrup & Asschenfeldt [4]	1979	9‑year‑old boy	Immunocompetent	Forearm	NR	Repeated surgical drainage	Recovered
Shaham et al. [8]	2023	10 children	Immunocompetent	Periorbital region	Culture-positive in 2/6 cases, unspecified	Incision + curettage + intra-op cryotherapy	All recovered
Present Case (2025)	2025	27‑year‑old male	Immunocompetent	Left inner thigh	Peptoniphilus asaccharolyticus	Surgical excision + metronidazole (post-C&S)	Recovered

This could be explained by the fact that there are two mechanisms of pathogenesis for molluscum-associated cutaneous abscesses. Either there could be a sterile abscess, wherein the inflammation is induced by the release of the molluscum bodies into the dermis, or there could be an additional superimposed bacterial infection. It is in the latter group of patients where additional antibiotics may improve the clinical outcome. The clinical features of infective versus sterile abscesses are similar, and it may not be possible to distinguish between these two on clinical grounds alone. Though infective abscesses tend to be much more tender than sterile abscesses in general, swabs should be taken from the discharge for culture and sensitivity, and the management should be tailored to the causative pathogen [4].

When treating anaerobic infections, antibiotics may be the only necessary therapy required, but surgical excision/revision may be a useful adjunct in case of severe infections not responding to antibiotics. *P. asaccharolyticus* is usually susceptible to penicillin, penicillin-tazobactam, amoxicillin-clavulanate, vancomycin, daptomycin, tigecycline, dalbavancin, carbapenems, ceftobiprole, linezolid, and metronidazole [15].

In our case, there was no response to amoxicillin-clavulanate in the initial stages, and the lesion progressed to an abscess; thus, surgical drainage of the left thigh abscess was performed with the surgical excision of the diseased tissue.

Both aerobic and anaerobic cultures were performed, and the only organism isolated was *P. asaccharolyticus,* which was found to be sensitive to metronidazole. Consequently, treatment with metronidazole was initiated, emphasizing the importance of combined surgical and antimicrobial intervention in deep-seated infections.

Learning points

Cutaneous abscess formation is a rarely reported complication of molluscum contagiosum in immunocompetent adults. Though usually reported to be a part of polymicrobial infections,* P. asaccharolyticus* could rarely be the only pathogen isolated in culture, as seen in our case. It is important to consider Gram-positive anaerobic cocci, including *P. asaccharolyticus,* as a potential pathogen in skin and soft tissue infections, especially in the setting of infections that are not responsive to conventional antibiotic therapies.

## Conclusions

This case highlights the importance of heightened clinical suspicion for secondary bacterial infections in chronic molluscum contagiosum (MC) lesions, even in the absence of trauma or immunosuppression. While MC is typically a benign and self-resolving condition, secondary infections can lead to significant complications. This report illustrates a rare presentation of an abscess caused by *Peptoniphilus asaccharolyticus*, an uncommonly recognized pathogen, necessitating both surgical and antimicrobial intervention. It underscores the role of anaerobic bacteria in soft tissue infections and emphasizes the need for targeted antibiotic therapy based on microbial culture results. Clinicians should remain vigilant for atypical presentations of MC, consider anaerobic cultures in non-resolving skin infections, and tailor antibiotic therapy accordingly.
